# Auswirkungen der Corona-Unterstützungsmaßnahmen auf die subjektive Gesundheit und das Wohlbefinden der Beschäftigten in staatlichen Hochschulen aus Sicht von Expert*innen

**DOI:** 10.1007/s11553-022-00986-6

**Published:** 2022-10-11

**Authors:** Arne Engelhardt, Sarah Hildmann, Marlena Löffler, Leonie Teichmann, Marlen Niederberger

**Affiliations:** Forschungsmethoden in der Gesundheitsförderung und Prävention, PH Schwäbisch Gmünd, Oberbettringer Str. 200, 73525 Schwäbisch Gmünd, Deutschland

**Keywords:** COVID-19, Hochschulen, Gesundheitsförderung, Nicht-wissenschaftliche Arbeitnehmende, Gleichstellung, Betriebliches Gesundheitsmanagement, COVID-19, Higher educational institutes, Health, Researchers/scientists/academics, Inclusion, Healthcare management

## Abstract

**Hintergrund:**

Die Coronapandemie und die notwendigen Maßnahmen zur Eindämmung des Infektionsgeschehens prägten in den Jahren 2020/2021 die Lebens- und Arbeitssituation aller Menschen. Auch deutsche Hochschulen mussten zur Fortführung der Forschung und Lehre Infektionsschutzmaßnahmen für Beschäftigte ergreifen. Diese hatten positive und negative Auswirkungen auf die subjektive Gesundheit und die Alltagsgestaltung der Beschäftigten. Um mögliche Gefährdungen zukünftig in ähnlich herausfordernden Situationen oder bei der Verstetigung von Maßnahmen zu vermeiden, ist die Kenntnis über gesundheitliche Auswirkungen von großer Bedeutung.

**Methode:**

Durch einen Mixed-Methods-Ansatz wurden Hochschulakteur*innen mit Expertise in den Bereichen Gleichstellung, Inklusion, Gesundheitsmanagement und Hochschulseelsorge mithilfe eines Online-Fragebogens zu den Auswirkungen von beruflichen Unterstützungsmaßnahmen im Zuge der Coronapandemie auf die subjektive Gesundheit und das Wohlbefinden der Beschäftigten in Hochschulen befragt.

**Ergebnisse:**

Insgesamt nahmen 117 Expert*innen an der quantitativen Befragung teil. Insbesondere die Entgrenzung zwischen Privat- und Berufsleben (71 %) sowie das Gesundheitsverhalten der Beschäftigten während der Pandemie (55 %) beurteilen diese kritisch. Den kollegialen Umgang zwischen Kolleg*innen nehmen die meisten (81 %) positiv wahr. Gleichzeitig sehen die Expert*innen eine Verschlechterung der sozialen Beziehungen (78 %). Ihrer Einschätzung nach ist die Arbeitssituation unter Coronabedingungen für die Beschäftigten überwiegend handhabbar (55 %), verständlich (71 %) und sinnvoll (64 %).

**Schlussfolgerung:**

Insgesamt zeichnen die Expert*innen ein differenziertes Bild über die Situation an den deutschen Hochschulen während der Coronapandemie. Es gibt Hinweise, dass sich die Situation für spezifische Beschäftigtengruppen in der Hochschule als deutlich herausfordernder erweist als für andere. Wird die Online-Lehre und Telearbeit an Hochschulen verstetigt, sind insbesondere Aspekte der sozialen Gesundheit und der Aspekt der Entgrenzung von Arbeit- und Privatleben differenziert nach unterschiedlichen Lebenslagen zu beachten.

## Abstract (en)

**Background:**

The coronavirus 2019 (COVID-19) pandemic and the necessary measures to contain the spread of the disease shaped the living and working situation of many people in 2020/2021. Universities also had to implement measures to protect employees from infection in order to continue research and teaching. These had positive and negative effects on the subjective health and everyday working situation of employees. In order to avoid possible risks in the future in similarly challenging situations or in the continuation of measures, knowledge about health effects is important.

**Methods:**

Using a mixed-methods approach, academic professionals and experts of equality, inclusion, health management, and counseling were surveyed using an online questionnaire on the impact of professional support measures in the wake of the COVID-19 pandemic on the subjective health and well-being of university employees.

**Results:**

A total of 117 experts participated in the quantitative survey. The dissolution of boundaries between private and professional life (71%) and the health behavior of employees (55%) were perceived critically. Most (81%) perceive the collegial interaction as positive. At the same time, the experts see a deterioration in social relations (78%). According to their assessment, the work situation under COVID-19 conditions is mostly manageable (55%), understandable (71%), and reasonable (64%) for the employees.

**Conclusion:**

The experts convey a differentiated picture of the situation. There is evidence that the situation is proving to be significantly more challenging for specific groups of employees in higher education than for others. If online teaching and telecommuting are to become established at universities, attention will have to be paid to aspects of social health and dissolution of boundaries between work and private life.

## Einleitung

Mittel- und langfristige Auswirkungen der Coronapandemie auf die Gesundheit der deutschen Bevölkerung sind im Jahr 2022 kaum einschätzbar, auch wenn klar scheint, dass die Folgen über Jahre oder Jahrzehnte spürbar bleiben werden [14, 16]. Der derzeitige Forschungsstand zu den gesundheitlichen Auswirkungen während der Pandemiezeit weist kritische, aber differenziert zu betrachtende Erkenntnisse auf.

Während zu Beginn der Pandemiezeit eine Reduktion des Stress- und Erschöpfungsgefühls [33] und ein Rückgang der Inanspruchnahme allgemein- und fachärztlicher Leistungen [9] registriert wird, nehmen im Laufe der Pandemiezeit die *negativen Folgen* auf die Gesundheit der Bevölkerung zu. Dies belegen die schlechteren Einschätzungen des subjektiven Gesundheitszustands und der allgemeinen Lebenszufriedenheit im Vergleich zu der Zeit vor der Pandemie, die gesteigerte Wahrnehmung von Einsamkeitsgefühlen sowie die leichte Erhöhung an depressions- und angstassoziierten Symptomen [13, 24]. Auch bei der physischen Gesundheit deuten die Erkenntnisse auf negative Folgen hin, insbesondere bei der Zunahme des Körpergewichts [18].

Dabei hängt die individuelle Gefährdung eng mit dem *sozialen Status bzw. der Lebenslage* zusammen [14, 18, 19, 33]. Negative gesundheitliche Folgen finden sich insbesondere bei Personen mit niedriger Bildung [18, 33], bei Alleinlebenden oder sozial benachteiligten Personen [14, 19] sowie bei Eltern, vorwiegend bei Müttern mit schulpflichtigen Kindern und bei Alleinerziehenden [3, 7]. Mütter von Kindern unter 6 Jahren erlebten einen starken Rückgang der allgemeinen Lebenszufriedenheit [7] und eine Zunahme des Erschöpfungs- und Stressgefühls [3, 31, 33]. Häufig verschwimmt im Rahmen von Homeoffice und Homeschooling zudem die Grenze zwischen Arbeits- und privater Zeit, was sich negativ auf die psychische Gesundheit auswirken kann [1, 7]. Gerade bei Beschäftigten mit jüngeren Kindern scheinen die Anforderungen von Care-Arbeit und Homeoffice während der Coronapandemie die Entgrenzung der Arbeits- und privaten Zeit zu verstärken [1, 16, 35].

Dies betrifft auch wissenschaftliche und nicht-wissenschaftliche Hochschulbeschäftigte mit Kindern. Auch sie arbeiten in der Pandemiezeit vermehrt im Homeoffice mit entsprechenden Entgrenzungstendenzen und gesundheitlichen Auswirkungen [20]. An einer Berliner Hochschule geben bspw. 71 % der Lehrenden an, einer starken allgemeinen Belastung ausgesetzt zu sein [4]. Auch international verzeichnen Studien eine Steigerung der physischen und mentalen Belastung, sowie die Verbreitung fundamentaler Ängste (z. B. Existenz- oder Infizierungsängste) bei Hochschulbeschäftigten [28, 38]. In einer englischen Studie geben 66 % der nicht-wissenschaftlich Mitarbeitenden ein hohes Stresslevel an [38]. In einer italienischen Studie zeigten 13 % der nicht-wissenschaftlichen Angestellten einen klinischen somatisch-affektiven Depressionswert [28]. Als Prädiktoren für die Vulnerabilität der Hochschulbeschäftigten erweisen sich insbesondere Geschlecht, Kinder und Statusgruppe [38]. Wissenschaftlerinnen mit jüngeren Kindern im akademischen Mittelbau haben weniger produktive Forschungszeit zur Verfügung und befürchten häufiger langfristige Folgen für die Karriere als ihre männlichen Kollegen [23, 32, 37]. Diese Befürchtung wird durch objektive Daten zur Einreichung und Veröffentlichung von Publikationen während der Pandemie gestützt [3, 39].

Die Erkenntnisse über die psychische Belastung und Beanspruchung von Beschäftigten in Hochschulen belegen die Relevanz psychischer Gefährdungsbeurteilungen auch in Pandemiezeiten sowie einer resilienten Hochschulpolitik, bei der Fragen der Gleichstellung und des betrieblichen Gesundheitsmanagements zusammengedacht werden [20]. Doch während der Coronapandemie führte die Mehrzahl der deutschen Hochschulen keine Gefährdungsprüfung durch [20], obwohl entsprechende Vorlagen und Empfehlungen vorlagen [5, 34]. Eine Befragung von Koordinator*innen im Gesundheitsmanagement gesundheitsfördernder Hochschulen deutet sogar auf einen pandemiebedingten Prioritätenverlust des Gesundheitsmanagements hin [22]. Gleichzeitig berichten über die Hälfte der befragten Hochschulleitungen beim Stimmungsbarometer 2020/21, dass sie sich während der Pandemie gut von der Politik unterstützt fühlten [36]. Inwieweit diese Einschätzung auch Hochschulakteur*innen mit Expertise im Bereich Gleichstellung, Chancengleichheit oder Gesundheitsmanagement in Hochschulen teilen und wie diese die Situation der Beschäftigten in der Coronazeit beurteilen, wurde im folgenden Forschungsprojekt untersucht. Dabei ging es um die Forschungsfrage: „Wie wirken sich die beruflichen Unterstützungsmaßnahmen in der Coronapandemie auf die subjektive Gesundheit und das Wohlbefinden der Beschäftigten in staatlichen Hochschulen aus?“

## Methodisches Vorgehen

In dem Forschungsprojekt wurden die Maßnahmen im Zuge der Coronapandemie an deutschen Hochschulen erfasst und die Auswirkungen auf die Beschäftigten aus Sicht von Expert*innen sowie der Beschäftigten untersucht. Integriert wurden Hochschulen aus Baden-Württemberg (BW) und Sachsen (SN). Die Auswahl der Bundesländer erfolgte aufgrund ihrer Position im Länderranking nach Gleichstellungsaspekten an Hochschulen: Baden-Württemberg und Sachsen bildeten im Jahr 2019 die Schlusslichter, weil sie bei Promotionen und Habilitationen sowie beim wissenschaftlichen Personal den niedrigsten Frauenanteil aufwiesen [27]. Zu vermuten ist, dass diesen Zahlen benachteiligende Strukturen zugrunde liegen, die auch während der Coronapandemie wirken und sich möglicherweise in dieser Zeit sogar verschärfen. Die Auswahl der Bundesländer Baden-Württemberg und Sachsen ermöglicht zudem beispielhaft die Unterscheidung zwischen Ost- und Westdeutschland. Zu vermuten war, dass sich kulturelle und institutionelle Unterschiede auf die Situation während der Coronapandemie auswirken. Beispielhaft seien hierfür die historisch begründeten Unterschiede hinsichtlich der Rolle der Frau in der Familie sowie der externen Betreuungsmöglichkeiten der Kinder genannt. Dieser Lesart zufolge wurde in Sachsen mit einer besseren Unterstützung der Familien als in Baden-Württemberg gerechnet. In beide Bundesländer hatte das Forscherteam zudem aufgrund vorhergehender Forschungsprojekte vielfältige Kontakte zu verschiedenen Praxispartner*innen, die über landesweite E‑Mail-Verteiler zu wichtigen Akteur*innen in den Hochschulen verfügten, was für die spätere Akquise der Befragten zentral erschien.

Im folgenden Artikel werden ausschließlich die Befunde der Expert*innenbefragung dargestellt. Für die Perspektive der Beschäftigten sei auf eine weitere Publikation verwiesen [2]. Als Expert*innen gelten Personen mit Expertise in den Bereichen betriebliches Gesundheitsmanagement oder Gleichstellung an Hochschulen. Einbezogen wurden Fachkräfte für Arbeitssicherheit, Betriebsärzt*innen von Hochschulen, Verantwortliche für betriebliches Gesundheitsmanagement, Gleichstellungsreferent*innen, Beauftragte für Chancengleichheit, betriebliche Berater*innen, Schwerbehindertenvertretung sowie Hochschulseelsorger*innen. Die Expertise in den Bereichen Gesundheit und Gleichstellung erschien relevant, weil die vorliegende Studienlage darauf hindeutet, dass insbesondere Mütter von der Coronapandemie negativ betroffen sind [17] und sich neben dem Risiko an COVID-19 („coronavirus disease 2019“) zu erkranken weitere gesundheitliche, aber auch berufliche Auswirkungen zeigen bzw. zukünftig zu erwarten sind [8, 33].

**Abb. 1:**
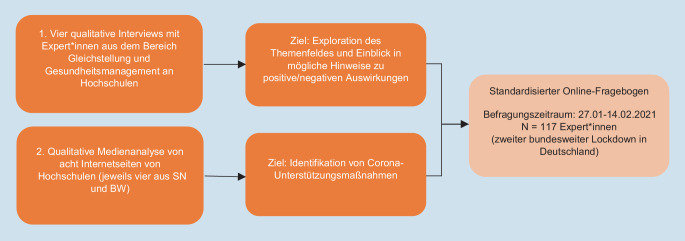
Studiendesign. *SN* Sachsen, *BW* Baden-Württemberg

Eingesetzt wurde ein exploratives sequenzielles Mixed-Methods-Design, bei dem mithilfe zweier qualitativer Vorstudien ein standardisierter Fragebogen entwickelt wurde (Abb. 1).

1. Zum einen wurden vier qualitative leitfadengestützte Expert*inneneninterviews über Zoom durchgeführt und aufgezeichnet. Ziel war es, die Erfahrungen der Expert*innen explorativ zu erfassen und ihre Eindrücke über die Gesundheit der Beschäftigten sowie der Unterstützungsmaßnahmen an Hochschulen zu erfassen. Die Expert*innenakquise erfolgte schriftlich per E‑Mail und adressierte Expert*innen aus Sachsen bzw. Baden-Württemberg, um für beide Bundesländer explorative Hinweise zu den Auswirkungen auf die Beschäftigten aus der Perspektive der Gleichstellung bzw. des Gesundheitsmanagements zu bekommen. Die Interviews wurden leitfadengestützt zwischen November 2020 und Januar 2021 mit vier Expert*innen (je zwei aus Sachsen und Baden-Württemberg) aus den Bereichen Gesundheit und Gleichstellung an Hochschulen geführt. Die Interviews wurden transkribiert und durch eine qualitative Inhaltsanalyse nach Mayring ausgewertet [29].
2. Zudem wurde im Zeitraum vom 20.12.2020 bis 04.01.2021 eine *Medienanalyse der Internetseiten von acht Hochschulen,* jeweils vier in Sachsen bzw. Baden-Württemberg, durchgeführt, um die Bandbreite an Unterstützungsmaßnahmen an Hochschulen zu identifizieren. Die Auswahl der Hochschulen erfolgte bewusst mit dem Ziel, möglichst unterschiedliche Hochschulen zu erhalten, bei denen aufgrund der Größe der Studiengänge sowie des Leitbildes vermutet werden kann, dass sie Coronamaßnahmen sehr umfassend und möglicherweise auch über die offiziellen Vorgaben hinaus umsetzen. Ausgewählt wurden daher Hochschulen mit einer hohen Anzahl an Studierenden sowie Hochschulen mit einem gesundheitsfördernden Leitbild und einer medizinischen Fakultät jeweils aus Baden-Württemberg und Sachsen. Die Internetseiten wurden ebenfalls mithilfe einer qualitativen Inhaltsanalyse ausgewertet [11] mit dem Ziel, einen explorativen Einblick in die Bandbreite an Unterstützungsmaßnahmen, auch über das vorgeschriebene Maß hinaus, zu bekommen.

Die Ergebnisse der qualitativen Vorstudien wurden diskursiv im Forscher*innenteam besprochen und zentrale Erkenntnisse, die in der bis dato vorliegenden Studienlage nicht thematisiert wurden, in den standardisierten Fragebogen aufgenommen. Von der Medienanalyse wurden vor allem die Unterstützungsmaßnahmen an Hochschulen und von den Expert*inneninterviews die herausfordernden Faktoren bei der Arbeitsgestaltung abgeleitet.

In diesem Artikel wird ausschließlich auf die dritte Teilstudie eingegangen, weil die Erkenntnisse der beiden qualitativen Vorstudien direkt in den Fragebogen eingeflossen sind.

### Erhebungsinstrument und Auswertung

Zur Entwicklung des Online-Fragebogens wurden neben den Erkenntnissen der vorangegangenen zwei Teilstudien der internationale Forschungsstand berücksichtigt, vorliegende Handlungsempfehlungen für Hochschulen während der Coronapandemie integriert [10, 12] und Praxispartner*innen aus den Bereichen Gleichstellung, Chancengleichheit und betriebliches Gesundheitsmanagement im Kontext Hochschule beteiligt (u. a. Koordinierungsstellen, die Unfallversicherungen). Sie haben bei der Entwicklung und dem Pretest des Fragebogens sowie bei der Rekrutierung der Teilnehmenden mitgewirkt.

Insgesamt beinhaltet der Fragebogen 20 geschlossene Items und 7 offene Fragen. Gegliedert ist er in 3 Abschnitte:

I. allgemeine Angaben zu der Expertise des/der Befragten,
II. Arbeitssituation für Beschäftigte an Hochschulen in der Zeit der Coronapandemie,
III. Bewertung der Unterstützungsmaßnahmen für die Beschäftigten an Hochschulen während der Coronapandemie.

Im Fragebogen wurde vorab geklärt, dass der Begriff Beschäftigte sowohl wissenschaftliches als auch nicht-wissenschaftliches Personal meint. Somit wurden die Expert*innen gebeten, die Situation aller Hochschulbeschäftigten einzuschätzen. Eine differenzierte Auswertung in wissenschaftliches und nicht-wissenschaftliches Personal ist dadurch nicht möglich. Alle Fragen beziehen sich auf den Zeitraum von März 2020 bis zur Durchführung des Online-Fragebogens im Februar 2021.

Es wurde der Befragungsserver SoSci Survey (SoSci Survey GmbH, München, Deutschland, Version 3.2.12) genutzt. Die Auswertung des Fragebogens erfolgte mit IBM SPSS® Statistics 26.0 (IBM Corp., Armonk, NY, USA). In der Auswertung wurden nur Fragebögen berücksichtigt, die zu mindestens 75 % (*n* = 117) ausgefüllt waren. Um signifikante Unterschiede zwischen Sachsen und Baden-Württemberg sowie zwischen den Expert*innen aus den Bereichen Gesundheit vs. Gleichstellung zu untersuchen, wurde der parameterfreie Mann-Whitney-*U*-Test durchgeführt. Signifikante Unterschiede werden referiert. Kommentare aus offenen Fragen wurden thematisch analysiert, mit dem Ziel zentrale Aspekte zusammenzufassen und zu systematisieren [6].

### Stichprobe und Rekrutierung

Die Rekrutierung der Experten*innen erfolgte durch eine Einladung per E‑Mail über den Zugang der Gatekeeper*innen. Die Gatekeeper*innen waren die Praxispartner*innen aus den Bereichen Gleichstellung und betriebliches Gesundheitsmanagement im Kontext Hochschule und Rektor*innen der Hochschulen aus Baden-Württemberg (BW) und Sachsen (SN). Die E‑Mail enthielt einen Abstract zur Studie, den Fragebogen als PDF-Version sowie einen Vorschlag für ein Anschreiben, um den Link des Online-Fragebogens an Expert*innen weiterzuleiten. Insgesamt wurden acht Praxispartner*innen beteiligt, die über landesweite E‑Mail-Verteiler in den Bereichen Gleichstellung, Inklusion, betriebliches Gesundheitsmanagement und Hochschulseelsorge verfügten.

**Abb. 2:**
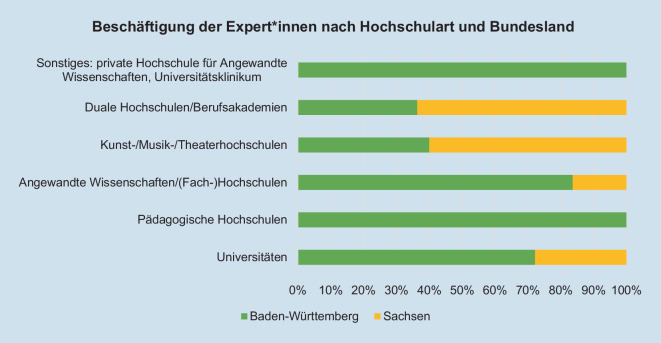
Beschäftigung der Expert*innen nach Hochschulart und Bundesland (*n* = 117; Mehrfachnennungen möglich)

An der Befragung nahmen insgesamt 150 Expert*innen teil. 16 Personen haben sich an der Befragung beteiligt, entsprechen aber nicht der zugrunde liegenden Expert*innendefinition. Zudem wurden 33 Befragte von der Auswertung ausgeschlossen, weil sie nicht mindestens 75 % des Fragebogens beantworteten. Somit wurden nach der Bereinigung des Datensatzes 117 Expert*innen berücksichtigt. Davon sind 28 Expert*innen aus Sachsen und 89 aus Baden-Württemberg (Abb. 2). Dies entspricht in etwa dem Verhältnis an Hochschulen in Sachsen (*n* = 30) und Baden-Württemberg (*n* = 80). 27 Expert*innen gehören nach eigenen Angaben dem Team eines Coronakrisenstabs bzw. -planungsstabs einer Hochschule an. Die Expert*innengruppe zeichnet sich durch Expertise in Gleichstellung und Gesundheit aus. Die Gleichstellungsexpert*innen umfassen 42 Beauftragte für Chancengleichheit/Gleichstellung/Familienfreundlichkeit und 19 Expert*innen einer Interessensvertretung wie z. B. Schwerbehindertenvertretung. Expertise im Bereich Gesundheit haben 4 Betriebsärzt*innen, 30 Fachkräfte für Arbeitssicherheit, 13 Verantwortliche für betriebliches Gesundheitsmanagement, 4 Hochschulseelsorger*innen und 5 Mitarbeitende einer psychosozialen Beratungsstelle.

## Ergebnisse

Im Folgenden werden die Ergebnisse der quantitativen Befragung der Expert*innen vorgestellt. Dabei werden die Arbeitssituation, die gesundheitlichen Auswirkungen, die beruflichen Herausforderungen, die Unterstützungsmaßnahmen sowie Empfehlungen zum Erhalt und zur Förderung der Gesundheit der Beschäftigten an Hochschulen während der Coronapandemie thematisiert.

### Arbeitssituation

**Abb. 3:**
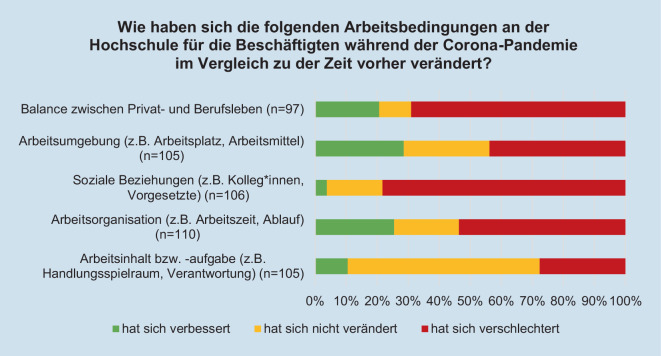
Veränderung der Arbeitsbedingungen der Beschäftigten an Hochschulen während im Vergleich zu vor der Coronapandemie

Insgesamt nehmen die meisten befragten Expert*innen eine Verschlechterung der Arbeitsbedingungen für die Beschäftigten während der Coronapandemie wahr (Abb. 3). Das betrifft insbesondere die Balance zwischen Privat- und Berufsleben, die Arbeitsumgebung, soziale Beziehungen sowie die Arbeitsorganisation. Arbeitsinhalt und -aufgabe blieben nach Ansicht der meisten Expert*innen während der Coronapandemie unverändert. Bei der Arbeitsumgebung zeigt sich, dass fast jede*r dritte Expert*in eine Verbesserung der Situation sieht.

**Abb. 4:**
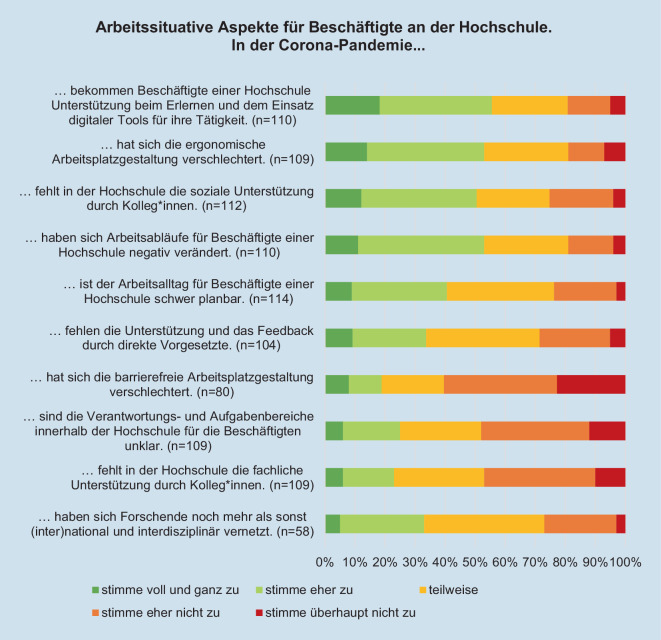
Arbeitssituation für Beschäftigte an der Hochschule während der Coronapandemie

Über die Hälfte der Expert*innen geben an, dass sich die Arbeitsabläufe der Beschäftigten *negativ *verändert haben (Abb. 4). 40 % der Expert*innen meinen, dass der Arbeitsalltag in der Pandemiezeit schwer planbar sei. Zudem fehle den Beschäftigten in der Hochschule die soziale Unterstützung durch Kolleg*innen (50 %). Den fehlenden fachlichen Austausch mit Kolleg*innen und direkten Vorgesetzen sehen dagegen nur 23 % bzw. 36 % der Befragten.

*Positiv *nimmt über die Hälfte der befragten Expert*innen die Unterstützungsangebote beim Erlernen und dem Einsatz digitaler Tools wahr. Dieser Aussage stimmen eher die Expert*innen aus dem Bereich Gesundheit zu (Mittelwert [MW] = 2,2 und Expert*innen Gleichstellung MW = 3,8, Mann-Whitney-*U*-Test [U] = 1142,5; *p*-Wert [*p*] = 0,002). Auch sieht jede*r dritte befragte Expert*in in der Coronapandemie die Chance zur (inter)nationalen und interdisziplinären Vernetzung. Fast die Hälfte der Expert*innen (insbesondere aus dem Bereich der Gleichstellung) glauben, dass Verantwortungsbereiche innerhalb der Hochschule den Beschäftigten in der Coronazeit klar waren (Expert*in Gleichstellung MW = 3,5; Expert*in Gesundheit MW = 3,0; U = 1158,0; *p* = 0,04).

### Gesundheitliche Auswirkungen

**Abb. 5:**
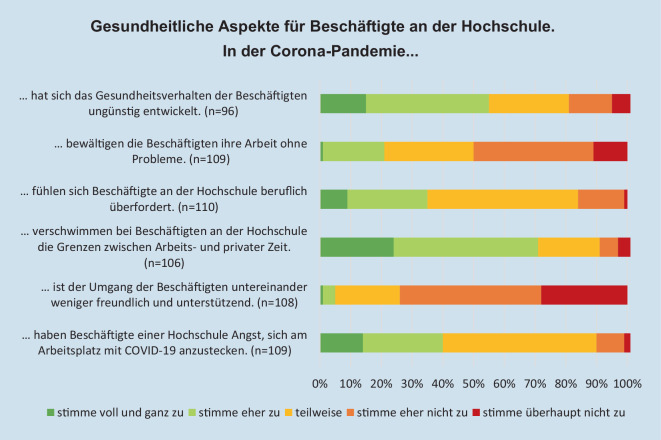
Gesundheitliche Aspekte für Beschäftigte an der Hochschule während der Coronapandemie

Die Expert*innen sehen negative *gesundheitliche Auswirkungen *auf die Beschäftigten beim Gesundheitsverhalten (55 %; Abb. 5). 71 % der Expert*innen berichten von einem Verschwimmen der Grenzen zwischen Arbeitszeit und privater Zeit. Mehr als jede*r dritte Expert*in glaubt, dass sich Beschäftigte während der Coronazeit beruflich überfordert fühlen. Die Hälfte glaubt nicht, dass die Beschäftigten ihre Arbeit ohne Probleme bewältigen können. Nur wenige Expert*innen (5 %) haben den Eindruck, dass sich der Umgang unter den Beschäftigten weniger freundlich und unterstützend entwickelt hat. Insbesondere die Expert*innen aus dem Bereich Gleichstellung sehen hier seltener eine negative Entwicklung (Expert*in Gleichstellung MW = 4,2; Expert*in Gesundheit MW = 3,8; U = 1142,5; *p* = 0,04).

**Abb. 6:**
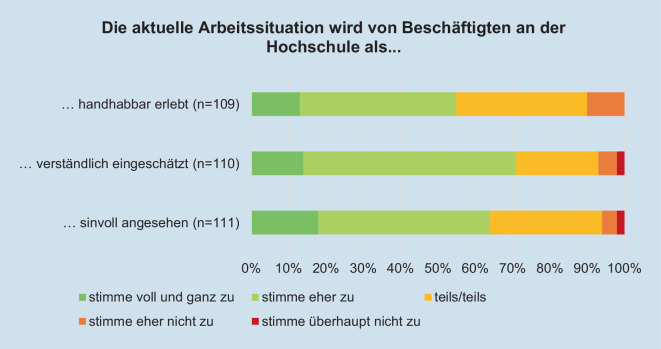
Beurteilung der Arbeitssituation von Beschäftigten an der Hochschule während der Coronapandemie

Die Expert*innen wurden nach ihrer Einschätzung zur Kohärenz der Beschäftigten befragt. Mehr als die Hälfte der Expert*innen nehmen ein hohes Kohärenzgefühl wahr, da die Beschäftigten die aktuelle Arbeitssituation an der Hochschule als handhabbar, verständlich und sinnvoll erachten (Abb. 6).

### Berufliche Herausforderungen

Die Expert*innen wurden auch gebeten, potenziell *herausfordernde Faktoren* für die Beschäftigten zu bewerten. Gemessen am Mittelwert sehen die Expert*innen folgende Herausforderungen, sortiert von der größten zur kleinsten (Skala 1 „sehr herausfordernd“ bis 5 „nicht herausfordernd“):

- Balance von Beruf- und Privatleben (MW = 2,1; Standardabweichung [SD] = 1,1; *n* = 110),
- Austausch mit Studierenden (MW = 2,1; SD = 1,0; *n* = 110),
- Austausch mit Kolleg*innen innerhalb der Hochschule (MW = 2,3; SD = 1,0; *n* = 114),
- Arbeitsverdichtung und lange Arbeitszeiten (MW = 2,5; SD = 1; *n* = 106),
- gegenseitige Wertschätzung und Anerkennung der geleisteten Arbeit zwischen den Beschäftigten (MW = 2,6; SD = 1,1; *n* = 106),
- Austausch und Vernetzung mit externen Kooperationspartner*innen (MW = 2,9; SD = 1,1; *n* = 104),
- Kurzarbeit bzw. Unterbeschäftigung (MW = 4,0; SD = 1,1; *n* = 92),
- Arbeitsplatzunsicherheit (MW = 4,2; SD = 1; *n* = 113).

Die Expert*innen aus dem Bereich Gleichstellung sehen die Balance zwischen Berufs- und Privatleben als deutlich herausfordernder an als die Expert*innen aus dem Bereich Gesundheit (Expert*in Gleichstellung MW = 1,8; Expert*in Gesundheit MW = 2,3; U = 1151,5; *p* = 0,025).

### Unterstützungsmaßnahmen der Hochschulen

**Abb. 7:**
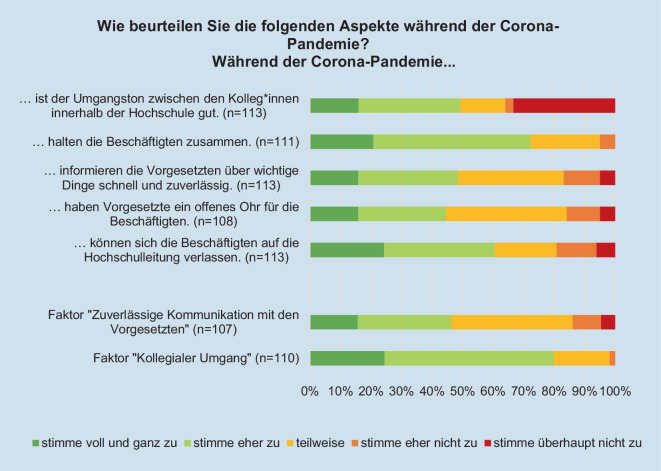
Beurteilung der Aspekte während der Coronapandemie

Die Hochschulen bieten und planen zum Befragungszeitraum verschiedene Unterstützungsformate für die Beschäftigten. Hierzu gehören nach den Angaben der Expert*innen Informationsschreiben zu aktuellen Entwicklungen und Maßnahmen (95 %; *n* = 111), die Flexibilisierung der Arbeitszeit (90 %; *n* = 106), Online-Schulungen/Weiterbildungen (83 %; *n* = 103), der Einsatz digitaler Austauschplattformen (75 %; *n* = 107), individuelle Beratungsangebote (70 %; *n* = 93) sowie die Berücksichtigung der Belange von Beschäftigten mit chronischen Erkrankungen und Behinderungen (69 %; *n* = 74). Seltener geben die Expert*innen an, dass es eine Unterstützung bei der Kinderbetreuung (46 %; *n* = 84), zum sozialen Austausch zwischen Kolleg*innen (46 %; *n* = 107), bei der Ausstattung des heimischen Arbeitsplatzes (35 %; *n* = 98), der temporären Verringerung der Arbeitszeit/des Lehrdeputats (27 %; *n* = 78) oder der Durchführung (psychischer) Gefährdungsbeurteilungen (21 %; *n* = 85) gibt. Die Expert*innen in Baden-Württemberg (BW) geben signifikant häufiger Unterstützungsangebote im Bereich der Kinderbetreuung an als die Expert*innen aus Sachsen (SN) (BW MW = 1,9; SN MW = 2,3; U = 531,0; *p* = 0,038). Durch eine explorative Faktorenanalyse („maximum likelihood“ mit Varimax-Rotation) wurden die Faktoren „zuverlässige Kommunikation mit den Vorgesetzten“ und „kollegialer Umgang“ gebildet Der Bartlett-Test (χ^2^-Test[10] = 218,058; *p* = 0,000) und das „Kaiser-Meyer-Olkin Measure of Sampling Adequacy“ (KMO = 0,709) weisen darauf hin, dass sich die Variablen für eine Faktoranalyse eignen. Es konnten explorativ zwei Faktoren gebildet werden, die 69 % der Varianz aufklären. Der Faktor „zuverlässige Kommunikation mit den Vorgesetzen“ enthält die 3 Items „schnelle und zuverlässige Information der Vorgesetzten über wichtige Dinge“, „offenes Ohr der Vorgesetzten gegenüber den Beschäftigten“ sowie „Verlässlichkeit der Hochschulleitung gegenüber den Beschäftigten“. Der Faktor „kollegialer Umgang“ enthält die zwei Items „guter Umgangston zwischen den Kolleg*innen innerhalb der Hochschule“ und „Zusammenhalt zwischen den Beschäftigten“. Die beiden Faktoren „zuverlässige Kommunikation mit den Vorgesetzten“ und „kollegialer Umgang“ werden von den meisten Expert*innen positiv beurteilt (Abb. 7).

**Abb. 8:**
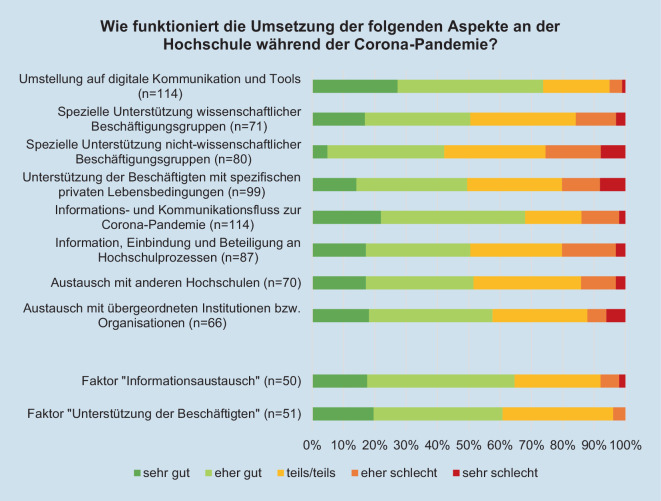
Beurteilung der Umsetzung der Aspekte während der Coronapandemie

Auch die Umsetzung verschiedener Unterstützungsmaßnahmen an der jeweiligen Hochschule wird überwiegend positiv bewertet (Abb. 8). So auch die beiden Faktoren „Unterstützung der Beschäftigten“ und „Informationsaustausch“, die das Ergebnis einer explorativen Faktorenanalyse („maximum likelihood“ mit Varimax-Rotation) sind. Der Bartlett-Test (χ^2^-Test [28] = 145,228, *p* = 0,000) und das KMO = 0,818 weisen darauf hin, dass sich die Variablen für eine Faktoranalyse eignen. Es konnten explorativ zwei Faktoren gebildet werden, die 61 % der Varianz aufklären. Der Faktor „Unterstützung der Beschäftigten“ enthält die vier Items „Umstellung auf digitale Kommunikation und Tools“, „spezielle Unterstützung der wissenschaftlichen Beschäftigungsgruppen“, „spezielle Unterstützung der nicht-wissenschaftlichen Beschäftigten“ sowie „Unterstützung der Beschäftigten mit spezifischen Lebensbedingungen“. Der Faktor „Informationsaustausch“ enthält die vier Items, „Austausch mit anderen Hochschulen“, „Austausch mit übergeordneten Institutionen bzw. Organisationen“, „Informationsfluss und Kommunikationsfluss zur Coronapandemie“ sowie „Information, Einbindung und Beteiligung an Hochschulprozessen“. Vergleichsweise kritisch wird die Unterstützung nicht-wissenschaftlicher Beschäftigungsgruppen und von Beschäftigten mit spezifischen privaten Lebensbedingungen gesehen (z. B. Zugehörigkeit zu einer gesundheitlichen Risikogruppe, Beschäftigte mit Kind/ern).

Eine explorative Missing-Analyse zeigt, dass insbesondere Expert*innen aus dem Bereich für Chancengleichheit/Gleichstellung/Familienfreundlichkeit und Interessensvertretungen (z. B. Schwerbehindertenvertretung) vor allem Fragestellungen zur speziellen Unterstützung von Beschäftigungsgruppen und dem Austausch mit anderen Hochschulen und Organisationen nicht beantworten. Zu vermuten ist, dass die genannten Expert*innengruppen wenig Einblick in diese Bereiche haben, weshalb sie diese spezifischen Fragen nicht beantworten können. In Bezug auf die Bundesländer lassen sich keine nennenswerten Unterschiede erkennen.

### Empfehlungen zum Erhalt und zur Förderung der Gesundheit aus Sicht der Expert*innen

Die Expert*innen wurden offen gefragt, ob Hochschulen auch nach der Coronapandemie Veränderungen brauchen, um die Gesundheit der Beschäftigten zu erhalten und zu fördern. Hier stimmen 94 % der Befragten zu. Bei den offenen Fragen werden insbesondere flexible Arbeitszeiten, die Möglichkeit zur Telearbeit und zu Online-Veranstaltungen in Forschung und Lehre angegeben (s. Infobox). Aber auch ein verstärkter Fokus auf die psychosoziale Gesundheit der Beschäftigten wird gefordert. Hierzu gehöre die Durchführung psychischer Gefährdungsbeurteilungen.

#### Infobox Kommentare der Expert*innen aus offenen Fragen

„Ja, Corona hat eine Sensibilität insbesondere auch für psychische Belastungen geschaffen, hier muss auch nach Corona ein Augenmerk draufgelegt werden.“ (Beauftragte*r für Chancengleichheit/Gleichstellung/Familienfreundlichkeit)

„Flexible/r Arbeitszeit und -ort … Schulung/Sensibilisierung für Achtsamkeit; Work-Life-Balance aufgrund des zunehmenden Verschwimmens der Grenzen.“ (Verantwortliche*r für betriebliches Gesundheitsmanagement)

„Beibehaltung des teilweisen Homeoffice, Beibehaltung der teilweisen digitalen Lehre (Hybridlehre), Digitalisierung aller Arbeits- und Lernbereiche weiter ausbauen.“ (Beauftragte*r für Chancengleichheit/Gleichstellung/Familienfreundlichkeit)

„Gesundheit als Führungsziel – auf allen Ebenen!“ (Betriebsarzt/Betriebsärztin)

„Die Durchführung einer psychischen Gefährdungsbeurteilung sollte beschleunigt werden, dies ist noch nie an dieser Hochschule passiert, jetzt umso wichtiger.“ (Beauftragte*r für Chancengleichheit/Gleichstellung/Familienfreundlichkeit)

„Ausgleichsangebote für besondere Stresssituationen wie (gemeinsame) Bewegungspausen, PMR (progressive Muskelrelaxation) oder ähnliches.“ (Fachkraft für Arbeitssicherheit)

Als konkrete Maßnahmen zur Gesundheitsförderung empfehlen die Expert*innen Gesundheits- und Bewegungsangebote, die auch Räume zur Begegnung schaffen. Das Thema soziale Gesundheit wird aber selten expliziert.

## Diskussion

Insgesamt zeichnen die Expert*innen ein differenziertes Bild über die Situation an den Hochschulen während der Coronapandemie ab. Die Hochschulen setzen die Coronamaßnahmen um, informieren die Beschäftigten, unterstützen beim Einsatz digitaler Tools und der Umgang untereinander wird mehrheitlich als kollegial wahrgenommen. Die Situation sei für die Beschäftigten verstehbar, handhabbar und sinnvoll, was eine wichtige Voraussetzung für die Gesundheit im Arbeitskontext ist [30]. Insgesamt deuten die Befunde an, dass bestehende Empfehlungen zur praktischen Umsetzung von Schutz- und Präventionsmaßnahmen in Hochschulen [34], mitunter auch über das erforderliche Maß umgesetzt werden.

Kritisch beurteilen die befragten Expert*innen insbesondere die Entgrenzung zwischen Privat- und Berufsleben sowie das Gesundheitsverhalten der Beschäftigten. Dabei ist zu beachten, dass diese beiden Punkte für den Hochschulkontext auch in Studien vor der Pandemie bereits als problematisch identifiziert wurden [26]. Hier zeigt sich auch die Notwendigkeit (psychischer) Gefährdungsbeurteilungen, die wie der Forschungsstand vor und während der Pandemie zeigt, selten an Hochschulen durchgeführt werden [15]. Hier ist insbesondere die Hochschulleitung in der Pflicht, auch wenn weitere Akteur*innen wie Führungskräfte, Betriebsärzt*innen, Fachkräfte für Arbeitssicherheit, Personalvertretung und betriebliches Gesundheitsmanagement einbezogen werden sollten.

Trotz der unter Coronabedingungen notwendigen räumlichen Distanz zwischen den Kolleg*innen und Vorgesetzten nehmen die Expert*innen den kollegialen Umgang zwischen Kolleg*innen als positiv sowie die Kommunikation mit den Vorgesetzten als zuverlässig wahr. Zugleich sehen viele eine Verschlechterung der sozialen Beziehungen, möglicherweise weil sich der Austausch auf die rudimentären, absolut notwendigen beruflichen Aspekte konzentriert. Zudem fehlt die soziale Unterstützung zwischen den Kolleg*innen. Sollte sich Online-Lehre und Telearbeit an Hochschulen dauerhaft etablieren, wie es sich die Expert*innen wünschen, sind die kurz- und mittelfristigen Effekte auf die soziale, psychische und physische Gesundheit der wissenschaftlichen und nicht-wissenschaftliche Beschäftigten weiter zu beobachten. Selbiges gilt für die Folgen für den weiteren Karriereverlauf von wissenschaftlichen Mitarbeiter*innen.

Negative Auswirkungen der Unterstützungsmaßnahmen auf die subjektive Gesundheit ergeben sich vor allem aufgrund der Veränderungen der Arbeitssituation. Insbesondere die Möglichkeit zum Homeoffice bzw. Telearbeit und die hohe Flexibilität erweisen sich in der Coronapandemie unter gesundheitlichen Aspekten als zwiespältig. Einerseits erlauben diese Maßnahmen einen gewissen Schutz vor einer COVID-19-Infektion, andererseits erscheint die damit einhergehende Entgrenzung des Arbeits- und Privatlebens sowie die soziale Isolation mitunter problematisch [25]. Bei einer Verstetigung von digitalen Angeboten und Strukturen an Hochschulen ist möglicherweise davon auszugehen, dass die ungünstigen Auswirkungen auf die subjektive Gesundheit zunehmen werden, wenn nicht entsprechend gegengesteuert wird. Wichtig erscheint es, dass Gleichstellung und Betriebliches Gesundheitsmanagement zukünftig verstärkt zusammenwirken, um die Bedarfe unterschiedlicher Beschäftigtengruppen unter struktureller und gesundheitlicher Perspektive umfassend, aber zielgruppengerecht, mitzudenken. Zielgruppenspezifische Unterstützungsmaßnahmen dienen der Bewältigung der beruflichen Herausforderungen und nehmen einen günstigen Einfluss auf die subjektive Gesundheit und das Wohlbefinden der einzelnen Beschäftigten.

In den Kommentaren der Expert*innen wird zudem deutlich, dass sich die Situation für spezifische Beschäftigtengruppen in der Hochschule als deutlich herausfordernder erweisen kann als die Gesamtergebnisse vermuten lassen. Verschiedene Studien belegen diesen Hinweis, indem Unterschiede zwischen männlichen und weiblichen Wissenschaftler*innen im Hinblick auf die Forschungsproduktivität und die Belastung während der Coronapandemie deutlich werden [23, 32]. So zeigt sich, dass in der Coronazeit überwiegend Frauen für Homeschooling, Haushalt und Kinderbetreuung während der Coronapandemie verantwortlich waren und entsprechend weniger Zeit für ihre Forschungen hatten [2, 21]. Auch die Spezifika von wissenschaftlich und nicht-wissenschaftlich Beschäftigten sind in weiteren Studien zu prüfen. Dies ist auch wichtig, weil sich bisherige Studien zu den Auswirkungen der Coronapandemie auf die Beschäftigten an Hochschulen eher auf Lehrende bzw. Wissenschaftler*innen konzentrieren [4].

Es konnten kaum signifikante Unterschiede zwischen Sachsen und Baden-Württemberg sowie zwischen den Expert*innen aus den Bereichen Gleichstellung und Gesundheit festgestellt werden. Dies deutet darauf hin, dass die Hochschulen in den beiden untersuchten Bundesländern ähnlich mit den Herausforderungen umgegangen sind. Eine differenzierte Analyse zwischen wissenschaftlichem und nicht-wissenschaftlichem Personal war aufgrund der Datengrundlage nicht möglich.

### Limitationen

Kritisch ist anzumerken, dass die Ergebnisse dieser Studie eventuell positiv verzerrt sind. Möglicherweise haben Rektor*innen und andere angesprochene Akteur*innen, die die Situation an ihrer Hochschule selbst kritisch sehen, den Aufruf zur Umfrage bewusst nicht weitergeleitet. Der Aufruf zur Befragung durch die Praxispartner*innen hatte den Vorteil, dass in kurzer Zeit viele Beschäftigte erreicht werden konnten, aber es kann keine Repräsentativität erreicht werden und es erlaubt keine Kontrolle des Rücklaufs. Eine Übertragbarkeit der Erkenntnisse auf andere Bundesländer oder auf einzelne Hochschulen ist nicht möglich. Zudem wird in diesem Artikel ausschließlich die Perspektive der Expert*innen und nicht die Sichtweise der Beschäftigten dargestellt.

Die Ergebnisse stellen eine Momentaufnahme im Kontext des zweiten bundesweiten Lockdowns dar. Im weiteren Verlauf der Coronapandemie wurden Bestimmungen und Verordnungen durchgehend an das Infektionsgeschehen angepasst, wodurch sich die Lage der Beschäftigten verändert hat und damit möglicherweise auch die Sichtweise der Expert*innen.

## Fazit für die Praxis

- Die Expert*innen kritisieren die zunehmende Entgrenzung zwischen Privat- und Berufsleben, welche sich bereits vor der Pandemie andeutete. Deswegen sollten die Mitarbeitenden von den Hochschulen über die Pandemie hinaus unterstützt werden, die Balance zwischen Privat- und Berufsleben zu vereinen.
- Entsprechend der Meinungen der Expert*innen wurden (psychische) Gefährdungsbeurteilungen vor und während der Coronapandemie selten an Hochschulen durchgeführt. Deshalb sollten Hochschulen dieser Thematik mehr Beachtung schenken.
- Ungünstige Auswirkungen auf die subjektive Gesundheit der Mitarbeitenden werden voraussichtlich durch die Verstetigung digitaler Arbeit an Hochschulen zunehmen. Zur zielgruppenspezifischen Unterstützung auf struktureller Ebene sollten das betriebliche Gesundheitsmanagement und Gleichstellung eng zusammenarbeiten.
